# Harnessing Physical Entropy Noise in Structurally Metastable 1T′ Molybdenum Ditelluride for True Random Number Generation

**DOI:** 10.1021/acs.nanolett.4c03957

**Published:** 2024-11-01

**Authors:** Yang Liu, Pengyu Liu, Yingyi Wen, Zihan Liang, Songwei Liu, Lekai Song, Jingfang Pei, Xiaoyue Fan, Teng Ma, Gang Wang, Shuo Gao, Kong-Pang Pun, Xiaolong Chen, Guohua Hu

**Affiliations:** † Department of Electronic Engineering, The Chinese University of Hong Kong, Shatin, New Territories, Hong Kong SAR 999077, China; ‡ Shun Hing Institute of Advanced Engineering, The Chinese University of Hong Kong, Shatin, New Territories, Hong Kong SAR 999077, China; § Department of Electrical and Electronic Engineering, Southern University of Science and Technology, Shenzhen 518055, China; ∥ School of Physics, Beijing Institute of Technology, Haidian, Beijing 100081, China; ⊥ Department of Applied Physics, Hong Kong Polytechnic University, Hung Hom, Kowloon, Hong Kong SAR 999077, China; # School of Instrumentation and Optoelectronic Engineering, Beihang University, Haidian, Beijing 100191, China

**Keywords:** true random numbers, molybdenum ditelluride, ferroelectric polarization, physical entropy noise, cryptography

## Abstract

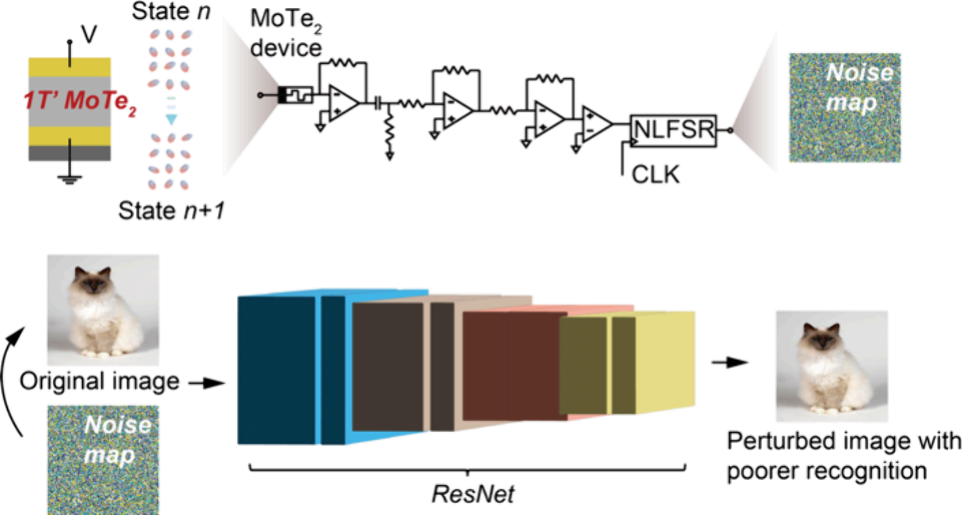

True random numbers are crucial for various research and engineering problems. Their generation depends upon a robust physical entropy noise. Here, we present true random number generation from the conductance noise probed in structurally metastable 1T′ molybdenum ditelluride (MoTe_2_). The noise, fitting a Poisson process, is proved to be a robust physical entropy noise at low and even cryogenic temperatures. Noise characteristic analyses suggest the noise may originate from the polarization variations of the underlying ferroelectric dipoles in 1T′ MoTe_2_. We demonstrate the noise allows for true random number generation, and this facilitates their use as the seed for generating high-throughput secure random numbers exceeding 1 Mbit/s, appealing for practical applications in, for instance, cryptography where data security is now critical. As an example, we show biometric information safeguarding in neural networks by using the random numbers as the mask, proving a promising data security measure in big data and artificial intelligence.

Random numbers, a string of random bits, play a crucial role in various research and engineering problems, for example, serving as the random inputs in numerical simulations and modeling, and introducing uncertainty in gaming and decision-making.^1^ However, the random numbers generated by the deterministic algorithms are essentially pseudorandom.^2^ As the demand for randomness is increasingly critical, the need for true random numbers has become pivotal.^3^ Physical entropy noise with inherent true randomness, such as thermal noise from thermally agitated charges and 1/*f* noise from charge trapping dynamics in semiconductors, can be employed for deriving true random numbers.^4^ However, arising from their physical nature, these physical entropy noises can be vulnerable to ambient noise and cryogenic attacks, undermining their reliability for true random number generation.^5^

Nanomaterials with a plethora of quantum phenomena present promising solutions.^6−8^ Among them, two-dimensional (2D) materials hold great interest, given their low-dimensionality and the quantum confinements, and the random variations exhibited in their underlying (opto)electronic and photonic processes.^9,10^ They can also give flexible integration and synergy with modern electronics.^11,12^ Indeed, reports show that the random dynamics in emerging 2D materials and devices can be employed as robust physical entropy noises for true random number generators (TRNGs) and physically unclonable functions (PUFs) toward data and hardware cryptography.^13−18^ 2D materials may even exhibit structural random variations. For example, molybdenum ditelluride (MoTe_2_), a transition metal dichalcogenide, can exist in a prismatic-orthorhombic (2H-1T) intermediate octahedral 1T′ phase.^19^ The intermediate phase can induce metastable polarization of the underlying ferroelectric dipoles and, as a result, random variations in the electronic structures and properties.^20^ Studies show the structural metastability, intrinsic to the 1T′ phase, is even resilient to ambient noise and cryogenic attacks,^21,22^ manifesting the potential of using 1T′ MoTe_2_ for true random number generation.

In this work, we report true random number generation from electrochemical-exfoliated 1T′ MoTe_2_. We show that the 1T′ MoTe_2_ allows stable conductance noise probing and, notably, fitting a Poisson process, the noise is proved a robust physical entropy noise even at low temperatures down to 15 K. Noise characteristic analyses, including spectral density and statistical time-lag, suggest the noise may arise from the polarization variations of the underlying ferroelectric dipoles in the 1T′ MoTe_2_. Using the noise, we realize true random number generation, and prove high-throughput secure random numbers exceeding 1 Mbit/s, appealing for practical applications in, for instance, cryptography. As an example, here we show a safeguarding measure of neural networks by masking key biometric information with the random numbers, promising for data security in big data and artificial intelligence.

MoTe_2_ predominantly exists in a stable 2H phase (Figure 1a).^23^ The 2H phase, however, can have local lattice distortions along the *y*-axis, with the Te atoms forming an octahedral coordination around the Mo atoms, leading to phase transition to a structurally metastable 2H-1T octahedral ferroelectric 1T′ phase.^23,24^ 1T′ MoTe_2_ can be produced via physical and chemical engineering processes.^25,26^ Toward scalable applications, here we adopt the electrochemical exfoliation method (Figure 1b; Supplementary Note 1).^27^ Briefly, tetrahexylammonium cations are used to intercalate the bulk MoTe_2_, leading to exfoliation and distortion of the 2H structure. Figure 1c presents a solution of the exfoliated MoTe_2_. Transmission electron microscopic (TEM) images of the nanosheets (Figure 1d,e) show the MoTe_2_ is successfully exfoliated with minimal defects. Particularly, a lattice spacing of 3.4 Å and a nonhexagonal structure are revealed, indicating the exfoliated MoTe_2_ is in the 1T′ phase.^28^ Electron diffraction (Figure 1e inset) proves a nonhexagonal rhombic, tetragonally symmetric lattice, confirming the 1T’′ phase. To verify the minimal defects, we perform X-ray photoelectron spectroscopic (XPS) analysis and prove a ∼ 1:2 ratio for the Mo and Te atoms (Figure S1). The minimal defects are ascribed to the tetrahexylammonium cation intercalation as the large molecules can effectively expand MoTe_2_ while limiting the intercalations.^27^ The minimal defects suggest the 1T′ phase transition primarily arises from the intercalation-induced lattice distortion rather than the defects.

**Figure 1:**
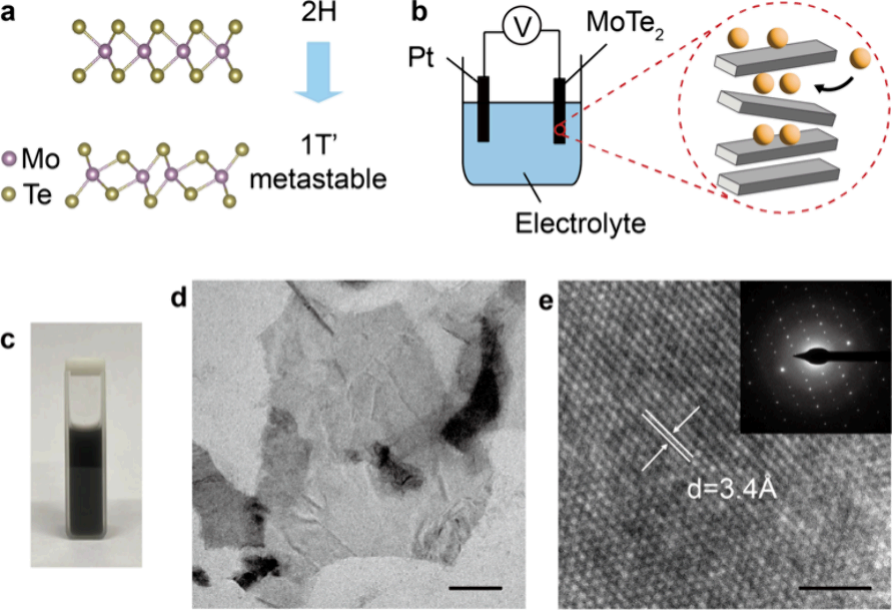
1T′ MoTe_2_ by electrochemical exfoliation. (a) Crystalline structures of hexagonal 2H and distorted octahedral 1T′ MoTe_2_. (b) Schematic electrochemical exfoliation of MoTe_2_, showing intercalation of tetrahexylammonium cations between the MoTe_2_ layers. Pt and MoTe_2_ are used as the electrodes. (c) Dispersion of the exfoliated MoTe_2_. (d, e) Transmission electron microscopic images and the selected electron diffraction pattern (inset of e) of the exfoliated MoTe_2_ nanosheets, proving a distorted octahedral 1T′ crystalline structure.

The structural metastability in the 1T′ phase may lead to random variations in the electrical properties. To probe the variations, we fabricate devices in a simple vertical structure, where the 1T′ MoTe_2_ is sandwiched between top and bottom electrodes (Figure S2; Supplementary Note 1). Indeed, as presented in Figure 2a, the device shows resistance switching (switching ratio >10^3^) with random variations under sweeping bias, proving a randomly varying electrical conductivity of the 1T′ MoTe_2_. The switching on the other hand may suggest a crystalline structure distortion toward the metallic 1T phase^26^ and/or a potential Stark modulation of the conductivity.^29,30^ Nevertheless, the random variations may be harnessed as a physical entropy noise. For convenient operation, we measure the current output from the 1T′ MoTe_2_ devices under static bias instead of sweeping bias, and probe the random variations exhibited in the output.

**Figure 2:**
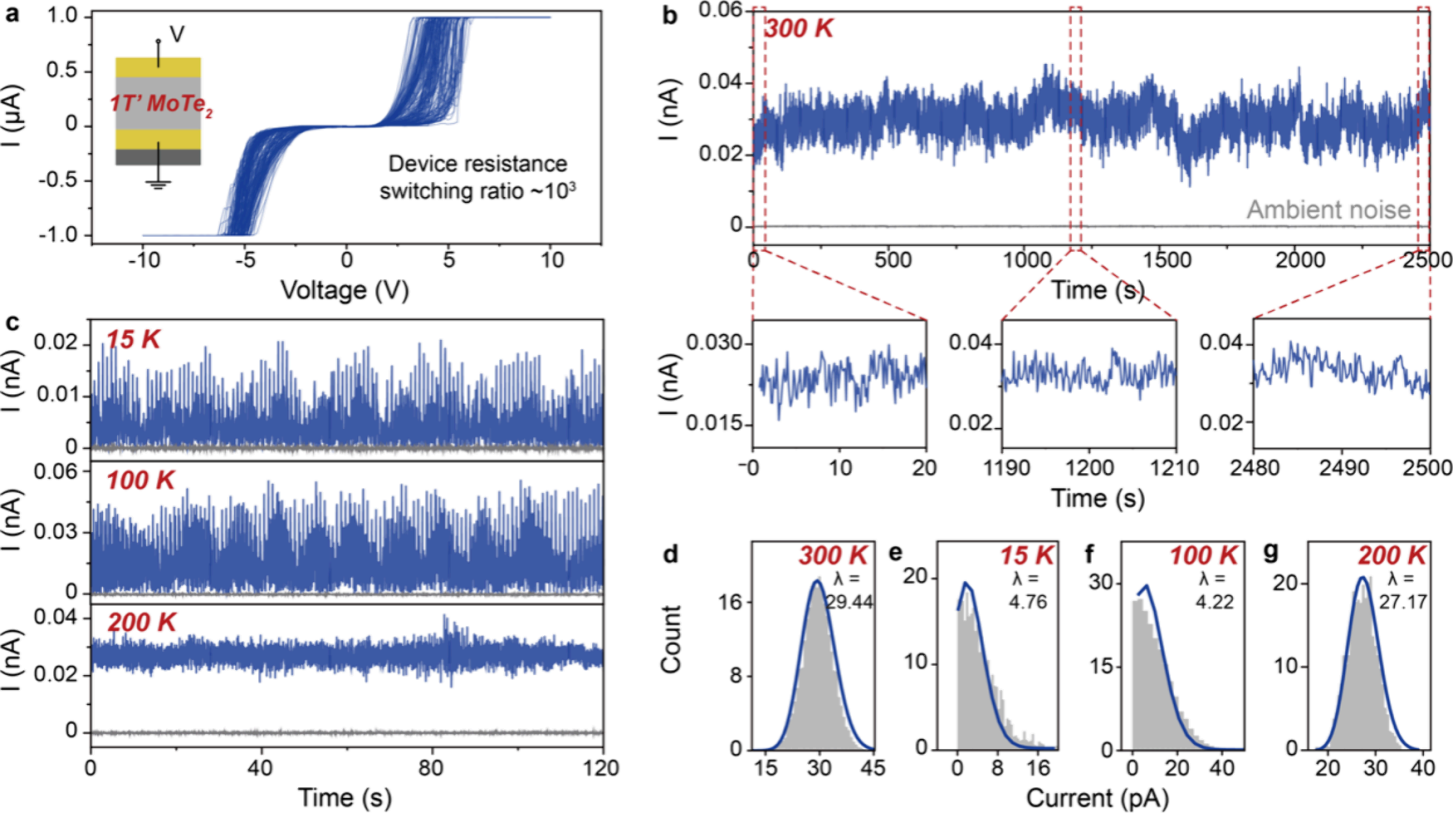
Conductance noise probing. (a) Current output for 100 sweeping test cycles of a typical 1T′ MoTe_2_ device, showing random variations in the device resistance switching at ∼5 V. The switching ratio is estimated as 1424 between the averaged high and low resistances. The inset shows the device structure. (b) Current output from the device at 300 K, showing stable conductance noise probed from 1T′ MoTe_2_. (c) Current output at low temperatures, showing stable conductance noise. The bias is 0.05 V for all the tests. See Figure S3 for the output at the other bias conditions. Ambient noise measured from suspended probing electrodes is presented in gray for comparison. (d–g) Histograms and Poisson fittings of the current output from (b) and (c), proving the conductance noise probed is a random process.

We first study the output at 300 K and 0.05 V (Figure 2b). See also Figure S3a–h for the outputs at 0.1–5 V. As observed, stable variations are demonstrated in the outputs at all the bias conditions. For simplicity, we refer to the variations as conductance noise. We plot histograms of the outputs and fit Poisson process distributions, as shown in Figure 2d (see also Figure S3i–p). Note the outputs also conform to normal distributions (Figure S4). Poisson process fitting is studied here to assess the randomness. A Poisson process describes a system of objects randomly distributed with an essential feature where the objects occur independently of one another.^31^ As such, the Poisson process fitting proves the conductance noise is a reliable random noise, i.e. a physical entropy noise.^32^ Given the request of the true random number applications for sustaining cryogenic attacks, we extend our assessment to low and even cryogenic temperatures (Figure 2c). As shown, stable conductance noise is proved at all the low temperatures, and the outputs can fit Poisson processes (Figure 2e–g). We note the Poisson fittings at the varying temperatures develop essentially consistent fitting characteristics, and that a larger λ at the higher temperatures means only a larger averaged number of events occurring per interval.^31^ Nevertheless, our investigation proves the conductance noise is a robust physical entropy noise stable at low and even cryogenic temperatures.

As discussed, noise from thermal and ambient electronics stands as a key source of physical entropy noise.^4^ We concurrently test the ambient noise to study whether the conductance noise is from thermal and/or the electrical test set-ups. As shown in Figure 2b,c, the ambient noise is smaller than the conductance noise by several orders of magnitude, proving the conductance noise is primarily from the 1T′ MoTe_2_ rather than the ambient noise. We also test the device-to-device and batch-to-batch conductance noises (Figures S5 and S6). From the tests, we learn that the device fabrication approach is reliable, with an acceptable yield in the initial trials (∼70–80%), and that the measured conductance noises all can well fit Poisson processes, though the outputs may vary in the current values. Further materials and device engineering are required to improve the device fabrication yield and uniformity.

To study the origin of the conductance noise, we perform current power spectral density (PSD) testing of the 1T′ MoTe_2_ devices at different bias and temperature conditions (Figure 3a–d, Figure S7). As shown, 1/*f* noise is proved over a broad frequency. For example, at 0.05 V, 300 K, the noise spectral power well fits 1, i.e. γ ∼ 1, proving a 1/*f* noise (Figure 3c). However, as the frequency increases, the output may be flattened at low bias conditions, indicating thermal noise dominates the high-frequency region (Figure 3d). The flattening may be ascribed to insufficient current signals at the low bias conditions.^33,34^ Nevertheless, 1/*f* noise is proved for our 1T′ MoTe_2_ devices, and this suggests charge fluctuations account for the conductance noise, for instance, charge trapping dynamics at the defect sites^35,36^ in our 1T′ MoTe_2_. However, as observed, varying the bias and temperature does not really variate the 1/*f* noise, suggesting charge trapping by defects may not account for the conductance noise. Indeed, as discussed, our 1T′ MoTe_2_ have minimal defects. We therefore understand the conductance noise arises from the intrinsic property of 1T′ MoTe_2_ rather than the defects. Given the structural metastability of the ferroelectric 1T′ phase, we assume the conductance noise is the reflection of ferroelectric polarization variations of the 1T′ MoTe_2_. However, ferroelectric polarization (Figure S8) and Raman spectroscopy (Figure S9) characterizations show no deterministic collective, overall ferroelectric polarization. The collective polarization may have been compromised by the discrete nature of the exfoliated 1T′ MoTe_2_ nanosheets,^37^ and further characterizations on the individual nanosheets are required to locate the exact polarization effect of the underlying ferroelectric dipoles. With the above investigations, we understand the conductance noise arises from the polarization variations of the ferroelectric dipoles in the individual 1T′ MoTe_2_ nanosheets.

**Figure 3:**
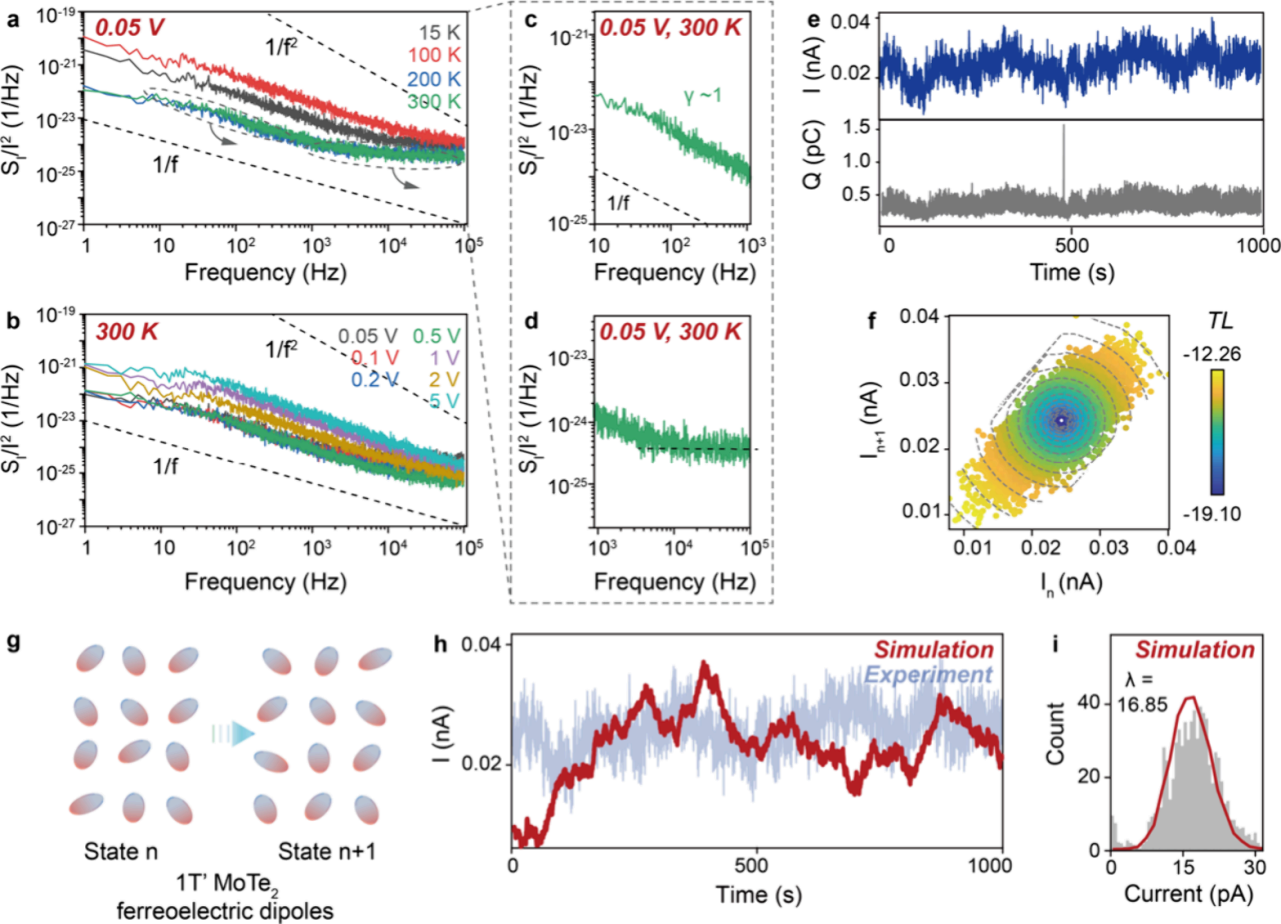
Origin of the conductance noise. Current power spectral density (PSD) of the 1T′ MoTe_2_ device at (a) 0.05 V and (b) 300 K, with (c, d) plotting PSD at low and high-frequency regions. PSD testing proves 1/*f* noise. (e) Current output and the corresponding cumulative charge fluctuation of the 1T′ MoTe_2_ device at 0.05 V, 300 K. The cumulative charge is integrated in the sampling interval of 0.067 s. (f) Time-lag plot for the cumulative charge fluctuation, showing bimodal distribution along the diagonal, suggesting stronger collations for the larger and smaller cumulative charge states to the next states. (g) Varying *n*-th and (*n*+1)-th polarization states of the ferroelectric dipoles in 1T′ MoTe_2_ in our proposed conductance noise mechanism. (h) Current output from Monte Carlo simulation on polarization variations of the ferroelectric dipoles, with the current output in (e) plotted as the background for comparison. (i) Histogram and Poisson fitting of the current output from Monte Carlo simulation.

To investigate the origin further, we study the cumulative charge characteristic of the devices. Here we plot in Figure 3e the output at 0.05 V, 300 K and the cumulative charge integrated during the sampling time intervals. The cumulative charge state can reflect the polarization state of the ferroelectric dipoles in 1T′ MoTe_2_.^38^ As observed, the cumulative charge proves a stable noise, indicating random variations in the polarization states. We adopt weighted time-lag, a method widely used for noise characteristic analysis,^39^ to statistically evaluate the noise. Briefly, as plotted in Figure 3f, the distribution of the cumulative charges is defined with a weighted time-lag
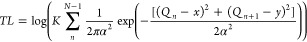
where *Q*_*n*_ and *Q*_*n*__+1_ are the *n*-th and (*n*+1)-th cumulative charge states, (*x,y*) denotes the corresponding *TL* plot coordinates, *N* is the total states, and α and *K* are the fitting parameters to ensure the *TL* maximum is 1 before logarithm. The cumulative charge states in the plot are distributed in ascending order, and a *TL* approaching 0 means a stronger correlation between the adjacent states. As observed, the *TL* plot shows random yet uniform aggregations with a bimodal distribution along the diagonal–both the larger and smaller cumulative charge states establish stronger correlations, while the medium cumulative charge states weaker correlations. This indicates the ferroelectric dipoles are uniformly distributed with bimodal aggregations, and that the strongly polarized dipoles may require a stronger current and a longer time to reverse.

We conduct Monte Carlo simulation on the polarization states of the ferroelectric dipoles in 1T′ MoTe_2_ (as illustrated in Figure 3g). See Supplementary Note 2 for the simulation method. Based on our understanding, the ferroelectric dipoles are uniformly distributed and undergo polarization variations. Under a static bias, the polarization variations can lead to fluctuations of the bound charges and thus, the conductance noise. As studied by our Monte Carlo simulation, the output renders a stable noise, consistent with our experimental results (Figure 3h); the conductance noise also well fits a Poisson process (Figure 3i). Therefore, the Monte Carlo simulation from the perspective of bound charge fluctuations aligns with our understanding that the conductance noise arises from the polarization variations of the ferroelectric dipoles.

See Figure S10 for the control experiments on the ambient noise and a MoS_2_ device, where we demonstrate the ambient noise and random telegraph noise develop distinctly different cumulative charge profiles and *TL* patterns. The comparison also aligns with our understanding that the conductance noise probed in 1T′ MoTe_2_ arises from the polarization variations of the underlying ferroelectric dipoles.

We design a very simple circuit in Cadence Virtuoso to harness the conductance noise for true random number generation (Figure 4a,b). The circuit consists of a 1T′ MoTe_2_ device, an I/V converter, a high pass filter, a voltage amplifier, and a comparator. Upon operation, the converter transforms the output from the 1T′ MoTe_2_ device into a voltage signal for convenient signal processing. The voltage signal, i.e. “*Output 1*” (Figure 4c), demonstrates a noise that can well fit a Poisson process (Figure 4d). This shows the circuit has well captured the conductance noise from the 1T′ MoTe_2_ device. The voltage signal then passes through the filter and amplifier to extract the noise in the form of differentiated voltage spikes, i.e. “*Output 2*” and “*Output 3*” (Figure 4c). As demonstrated, the voltage spikes also fit a Poisson process (Figure 4e,f), proving the extracted noise remains a robust physical entropy noise. The comparator then processes the voltage spikes to yield random numbers in the form of 0s and 1’s binary strings, i.e. “*Output 4*” (Figure 4c). See also Figure S11 for a zoomed-in distribution of the 0s and 1’s digits. As demonstrated, the random 0s and 1’s digits render a ratio of ∼1:1 (Figure 4g), suggesting the numbers are random.

**Figure 4:**
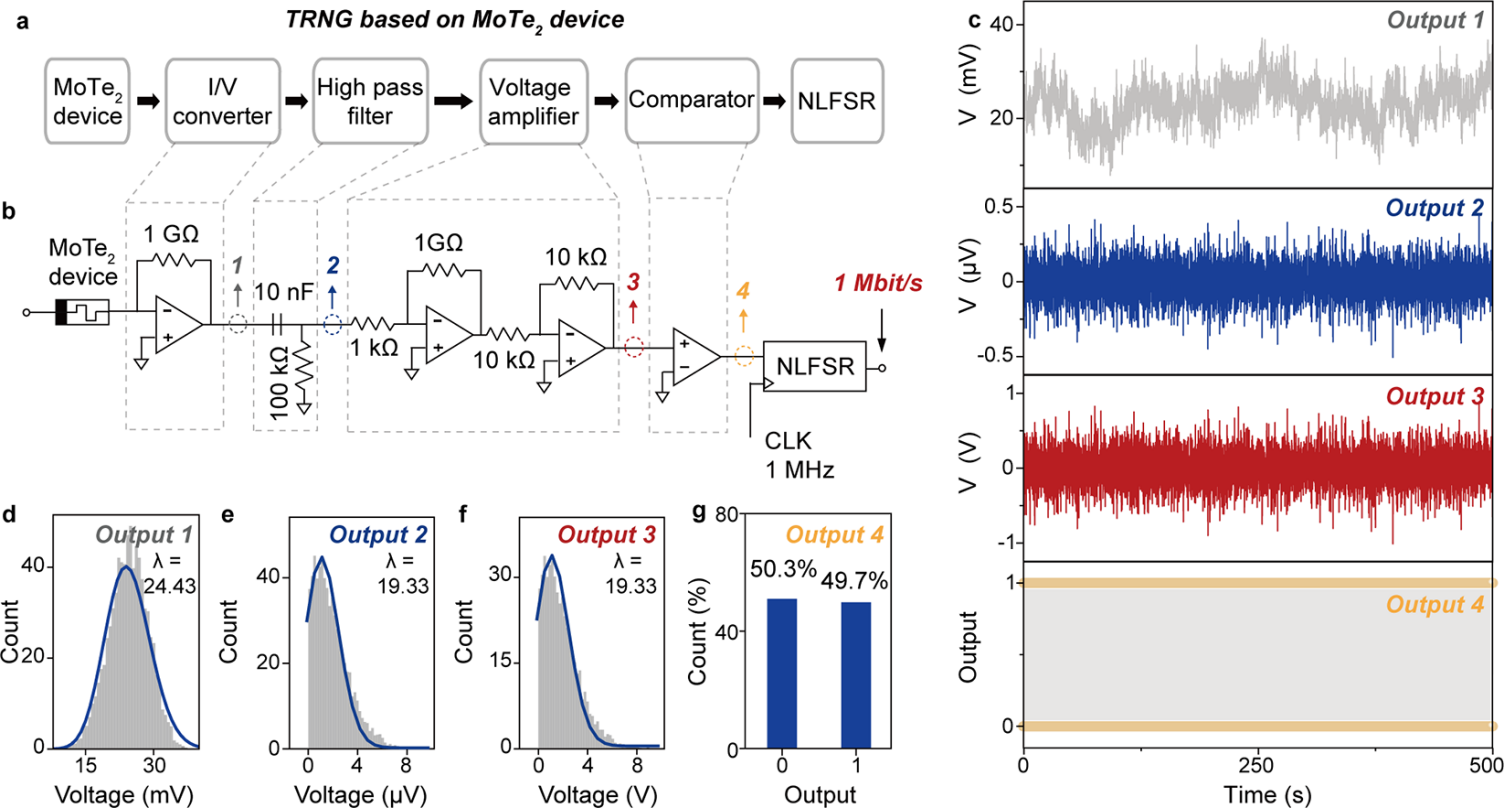
True random number generation. (a, b) Circuit design and diagram of the true random number generator (TRNG), consisting of the 1T′ MoTe_2_ device, I/V converter, high pass filter, voltage amplifier, comparator, and NLFSR. NLFSR (Figure S12) is used to generate high-throughput random numbers from the seed, i.e. the true random numbers from Port 4. (c) The output obtained from the TRNG at Port 1–4, including the converted voltage output, the filtered voltage output, the amplified voltage output, and the generated true random numbers in a string of 0s and 1’s. See Figure S11 for the 0s and 1’s string details. (d–f) Histograms and Poisson fittings of the data points from Output 1–3 in (c), proving all the outputs are random processes. Note absolute values are taken from the negative data points in Output 2 and 3 for the Poisson fittings. (g) Histogram of the 0s and 1’s true random numbers in Output 4 in (c), demonstrating a ratio of ∼1:1.

To verify the random numbers are truly random, we test the randomness using the National Institute of Standards and Technology (NIST) randomness testing suite that is widely used to evaluate the randomness.^40^ As shown in Table S1, the random numbers successfully pass the test, proving their true randomness. However, the throughput is ∼10 bit/s, limiting their use.

To address the limitation, employing a common approach in the field,^41^ we use the random numbers as the seed and introduce them to a Nonlinear Feedback Shift Register (NLFSR; Figure S12) for high-throughput random number output with a rate of, say, 1 Mbit/s (Figure 4a,b). Here, as a case example, we present in Figure S13a random number bitmap generated using the high-throughput random numbers. Notably, as shown in Table S2, the high-throughput random numbers also fully pass the NIST randomness test.

Here we note the electrical components (e.g., the resistors and comparators) in Figure 4b are configured to adapt to room temperature operation. For low temperature (e.g., 15 K) operations, the electrical components need to be reconfigured to adapt to low current values, and special temperature-compensating and corrective circuits may be used.^42^ Nevertheless, the demonstrated capability of our 1T′ MoTe_2_ devices for surviving cryptogenic attacks is critical for TRNG applications, outperforming the current TRNG techniques (Table S3).

The high-throughput secure random numbers are appealing for practical applications.^3^ As an example, we apply the high-throughput secure random numbers in cryptography. Cryptography is of critical importance in the current age with the data exponentially growing and at the risk of being attacked and sabotaged.^3^ See Figures S14 and S15 and Supplementary Movies S1–3 for demonstrations of password generation and data encryption.

Beyond the common cryptographic applications, the importance of cryptography is manifested by the rapid advances of neural networks. Neural networks are widely used in, for instance, image recognition, sensing, autonomous driving, and manufacturing, where sensitive data is constantly required. Particularly, in the context of big data and artificial intelligence, sensitive biometric information such as retinas, facial characteristics, and fingerprints is excessively used and potentially leaked.^43^ Secure data protection has therefore become a critical issue, and adversarial attacks in neural networks pose a significant threat.^43^ Here we adopt a differential safeguarding strategy (Figure S16) and investigate its effectiveness to obfuscate sensitive data in neural networks.^43^ The differential safeguarding framework injects random numbers as noise into the target data for perturbation. Following this approach, we train a residual neural network (*ResNet*) for pet recognition (Figure 5a; Supplementary Note 3).^44,45^*ResNets* are a widely used model for image and pattern recognition.^45^ After training, the model performs successful pet recognition with an accuracy of 92%. See the confusion matrix and performance of the well-trained model in Figure S17.

**Figure 5:**
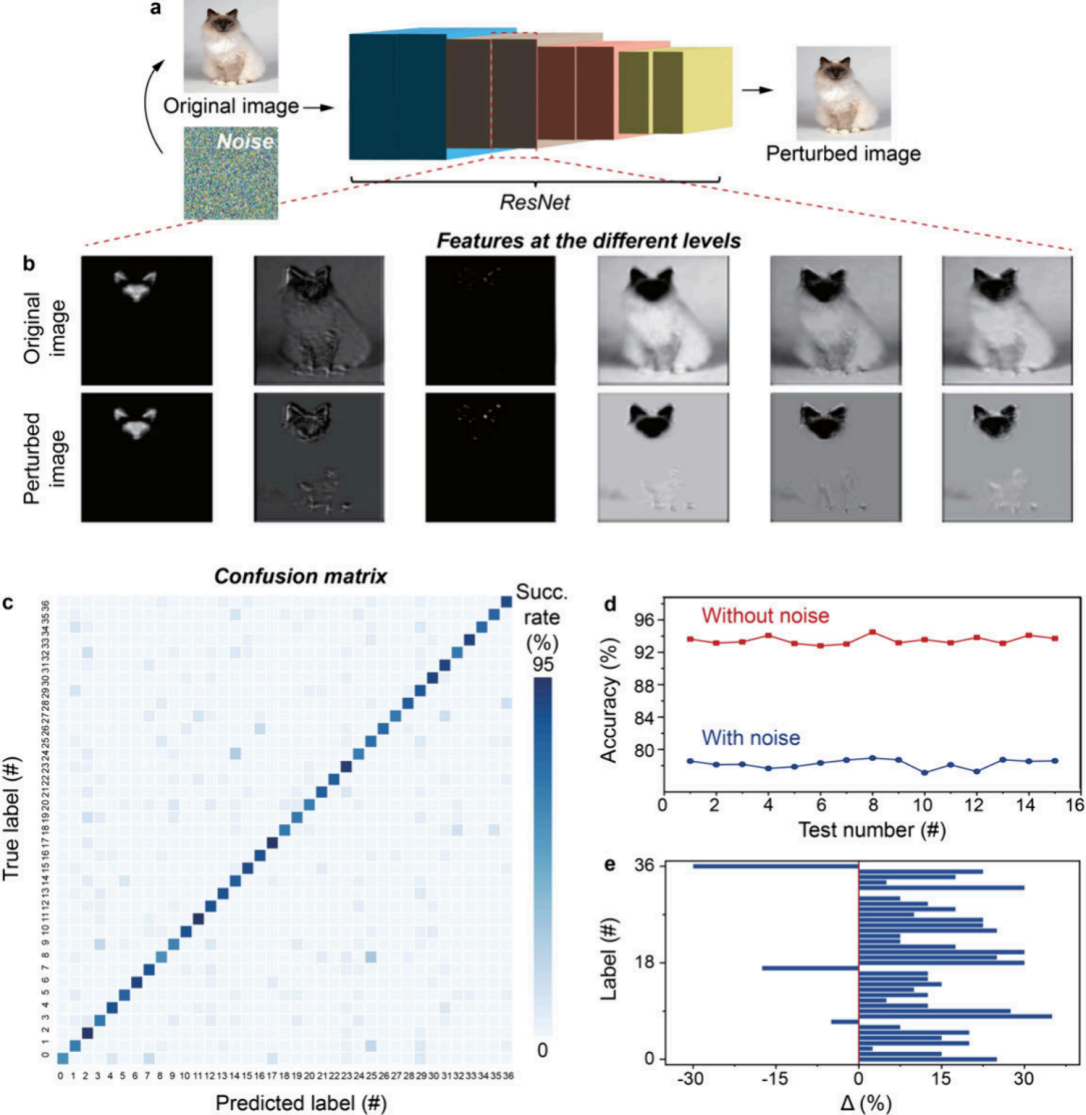
Safeguarding in neural networks. (a) ResNet variant architecture for cat (from Oxford-IIIT Pet Dataset^44^) recognition without and with noise perturbation. The noise map is produced using the high-throughput random numbers. (b) The cat images without and with noise perturbation at the intermediate convolution layers, showing the perturbed images lose certain features of the cat throughout the convolution layers. (c) Confusion matrix for the ResNet variant recognition with noise perturbation. The scale corresponds to the success rate of the predicted labels. The *x* and *y* coordinates denote the predicated and true labels of the 37 classifications in the training data set. (d) The accuracy with and without noise perturbation at the different test numbers. (e) The difference between the success rates in the confusion matrices with and without noise perturbation along the diagonal. The *x* coordinate denotes the difference in the success rate along the diagonal, and the *y* coordinate the 37 different classifications. The confusion matrix details with the success rate values are presented in Figure S18.

We then inject the random numbers as noise into the target validation data. Interestingly, as shown in Figure 5a, taking the image of a Siamese cat for demonstration, the noise perturbation appears negligible to human eyes. This is ascribed to the innate ability of the human brain to process visual information holistically, i.e. focusing on the broader picture rather than the minute details.^46^ However, as demonstrated in Figure 5b, the noise perturbation substantially affects the recognition of the model at all the convolution layers. The confusion matrix and accuracy (∼78%) demonstrate the *ResNet* variant with the noise perturbation gives a poor performance in recognition (Figure 5c,d). See also Figure S18 for the confusion matrix details with noise perturbation. Comparing the accuracies with and without the noise perturbation (Figure 5d), a bit noise perturbation can cause a substantial degradation in the classification accuracy. This is because the noise disrupts the feature detection capability of the *ResNet* variant in the initial layers, which then propagates to the deeper layers, leading to exacerbation of the errors.^47^ The detailed difference (i.e., Δ) between the two success rates in the different 37 categories can be found in Figure 5e. The findings prove that injecting the random numbers as noise perturbations that are not discernible to eyes can substantially interfere with the neural networks and as such, enhance data security in neural networks.

In this work, we have reported true random number generation using structurally metastable 1T′ MoTe_2_. Our analysis suggests the polarization variations of the ferroelectric dipoles in 1T′ MoTe_2_ give rise to a conductance noise, and that the noise can be harnessed as a robust physical entropy noise for true random number generation. Notably, the noise remains stable even at cryptogenic temperatures, critical for the cryptogenic applications of true random numbers. We have proved true random number generation and their use as the seed for high-throughput secure random number generation exceeding 1 Mbit/s, appealing for various practical applications, for instance, cryptography. As an example, we demonstrate data safeguarding in neural networks by using the random numbers as the mask. Neural networks pose a significant threat to data security, particularly the biometric information, in big data and artificial intelligence by adversarial data leakage and attacks. Our safeguarding approach can serve as a critical data security measure. Given this, and the scalability as well as seamless integration possibility with the electronics systems of the electrochemical-exfoliated 1T′ MoTe_2_, our approach of true random number generation holds great potential to enable secure data in neural networks.

## Data Availability

The codes for the Monte Carlo simulation, data encryption, and pet recognition are available from the corresponding author upon request.

## Supporting Information Available

Supporting Information, including experimental methods and supporting figures on exfoliation and device fabrication of 1T′ MoTe_2_, materials and device characterizations, Monte Carlo simulation, data encryption, and neural network data safeguarding. (PDF)
Demonstration of password generation (MP4)
Demonstration of video data encryption (MP4)
Demonstration of audio data encryption (MP4)
