# Supplementary material for: Harnessing Physical Entropy Noise in Structurally Metastable 1T′ Molybdenum Ditelluride for True Random Number Generation
Source: Nano Lett. 2024 Nov 1;24(45):14315–22. doi: 10.1021/acs.nanolett.4c03957 (PMC11565741; doi:10.1021/acs.nanolett.4c03957)
Supplement: Supplementary file 1 — nl4c03957_si_001.pdf [file nl4c03957_si_001.pdf]

SUPPORTING INFORMATION

Harnessing physical entropy noise in structurally metastable 1T' molybdenum ditelluride for true random number generation

Yang Liu[1,2], Pengyu Liu[1], Yingyi Wen[1], Zihan Liang[3], Songwei Liu[1], Lekai Song[1], Jingfang Pei[1], Xiaoyue Fan[4], Teng Ma[5], Gang Wang[4], Shuo Gao[6], Kong-Pang Pun[1], Xiaolong Chen[3], Guohua Hu[1,]*

[1]Department of Electronic Engineering, The Chinese University of Hong Kong, Shatin, New Territories, Hong Kong S. A. R., 999077, China

[2]Shun Hing Institute of Advanced Engineering, The Chinese University of Hong Kong, Shatin, New Territories, Hong Kong S. A. R., 999077, China

[3]Department of Electrical and Electronic Engineering, Southern University of Science and Technology, Shenzhen, 518055, China

[4]School of Physics, Beijing Institute of Technology, Haidian, Beijing, 100081, China

[5]Department of Applied Physics, Hong Kong Polytechnic University, Hung Hom, Kowloon, Hong Kong S. A. R., 999077, China

[6]School of Instrumentation and Optoelectronic Engineering, Beihang University, Haidian, Beijing, 100191, China

*Correspondence to: ghhu@ee.cuhk.edu.hk

Supplementary Notes

Supplementary Note 1: Material production and device fabrication

The raw $MoTe_2$ powder and other chemicals are purchased from Alpha Aeser and Sigma-Aldrich, and are used as received. The Raw $MoTe_2$ powder is pressed with high pressure into a solid disk for the electrochemical exfoliation. The electrochemical exfoliation follows the method reported in Ref. (1). For device fabrication, the Au/$MoTe_2$/Au devices are fabricated on Si/SiO_2, where $MoTe_2$ is deposited by spin-coating, and the gold electrodes are deposited by electron-beam evaporation. The electron-beam evaporator is IVS EB-600. The $MoTe_2$ after deposition is baked at 400 ^oC for 0.5 hours under nitrogen.

Tektronix Keithley 4200-SCS parameter analyzer is used to measure the electrical characteristics of the devices under 300 K at ambient conditions. For the 15 K, 100 K, and 200 K tests, FS-Pro is used under vacuum (~10^{-6} mbar).

Supplementary Note 2: Monte Carlo simulation

Assuming a constant electric field, the change in the polarization of the ferroelectric dipoles will not affect the field, but the internal polarization state of the ferroelectric dipoles in the 1T' $MoTe_2$ will change. The fluctuation of the polarization can lead to fluctuating bound charges, which can in turn cause fluctuations in the conductance state of the 1T' $MoTe_2$.

The polarization can switch between two states, i.e. P_1 and P_2, and the switching follows the Arrhenius law [2]. The polarization can thus be modelled as a Poisson process, meaning that the probability of a switch in a small interval of time dt is given by λ*dt, where λ is the rate of the process. The rate follows the Arrhenius law, given by $\lambda = \mathrm{A} * \exp(-\mathrm{E}/(\mathrm{k} * \mathrm{T}))$, where A is the pre-exponential factor, E is the energy barrier, k is the Boltzmann constant, and T is the temperature. However, the switch in polarization now results in fluctuations in the bound charge instead of a change in the electric field. The bound charge ρ_B is related to the polarization P by the relation $\rho_B = -divP$, where div is the divergence operator, indicating that the bound charge is related to the spatial variation of the polarization. In a simple one-dimensional case, this can be described as $\rho_B = -dP/dx$. Now assume that the change in polarization is uniform across the material, the bound charge will change by $\Delta\rho_B$ proportional to the change in polarization ΔP, expressed as $\Delta\rho_B = -\Delta\mathrm{P}/\mathrm{L}$, where L is a characteristic length scale of the system. This change in voltage can cause a current to flow.

We propose that the change in the bound charge affects the resistance R of the material. A simple model assumes that the resistance is inversely proportional to the absolute value of the bound charge, $\mathrm{R} = \mathrm{R}_0/\rho_B$, where R_0 is a constant initial resistance. Finally, apply a constant voltage V across this variable resistor, the current I through the material at any time would be given by Ohm's

law, $\mathrm{I(t) = V/R(t)}$. The charge from the current can be described by $Q_i = \int_{t=i}^{t=i+1} dI/dt$. So every time the polarization switches, it will change the bound charge, which will then change the resistance and hence, the current fluctuations.

Consider a common effect often found in materials known as Poole-Frenkel behavior [3], the current through the material (and hence the resistance) is affected by the applied electric field (which in our case can be linked to the bound charge), and the current density J is given by $\mathrm{J = J_0} * \exp(\beta * \sqrt{\varepsilon})$, where $\mathrm{J_0}$ is the current density at zero field, ε is the electric field, and β is a material constant. In our scenario, we now link the bound charge and the electric field ε. Assume that the change in the bound charge $\Delta\rho_B$ is proportional to the change in electric field $\Delta\varepsilon$, the bound charge is expressed by $\Delta\rho_B = -\Delta\varepsilon/L$. Then, the current density will be dependent on the bound charge. Note that the $sqrt(\varepsilon)$ means that this is not a simple linear or inversely proportional relationship.

Supplementary Note 3: Neural network recognition

The neural network security is carried out in Python 3 and is based on the *ResNet* framework. [4] The *ResNet* variant is based on a *ResNet* 34 structure, consisting of convolution layers, residual blocks, and so on. The detailed information about *ResNet* 34 can be found at: *https://pytorch.org/vision/main/models/generated/torchvision.models.resnet34.html*. The public dataset is from the Visual Geometry Group at the University of Oxford (available at *https://www.robots.ox.ac.uk/~vgg/data/pets/*). [5] The dataset consists of a 37-category pet dataset with roughly 200 images for each class with different scales, poses, and lighting. All the images have an associated ground truth annotation of breed, head ROI, and pixel-level trimap segmentation, and those are used for training and testing.

Supplementary Figures

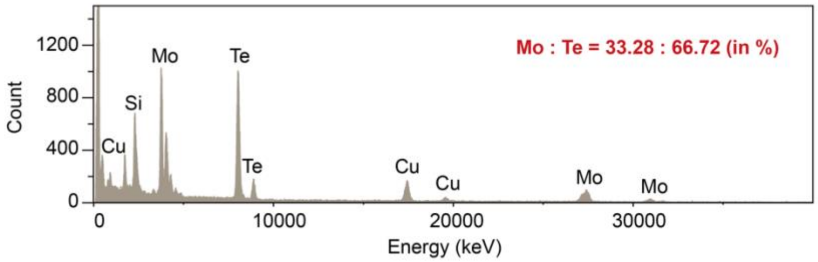

Figure S1. Energy dispersive X-ray spectrometry element mapping of the exfoliated 1T' $MoTe_2$ nanosheets. The ratio of the Mo and Te atoms is 33.28: 66.72 (in %), proving that there are minimal defects in the exfoliated 1T' $MoTe_2$ nanosheets.

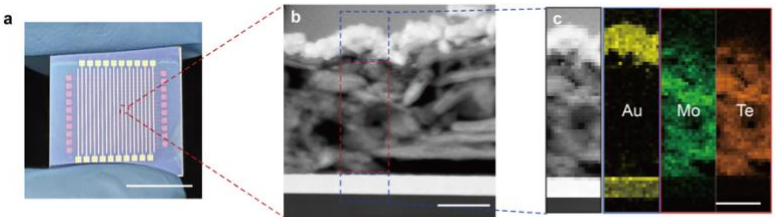

Figure S2. 1T’ $MoTe_2$ device array. (a) Image of an array of 20 ×20 1T’ $MoTe_2$ devices. Spin-coated 1T’ $MoTe_2$ is sandwiched between the evaporated top and bottom gold electrodes to fabricate the devices. The substrate is Si/SiO_2. (b) Cross-sectional scanning electron microscopic image of a typical device, and (c) the corresponding elemental analysis of the selected areas for the Au, Mo, and Te elements, showing clear interfaces between the $MoTe_2$ layer and the electrodes. Scale bars – (a) 1 cm, (b) 300 nm, and (c) 300 nm.

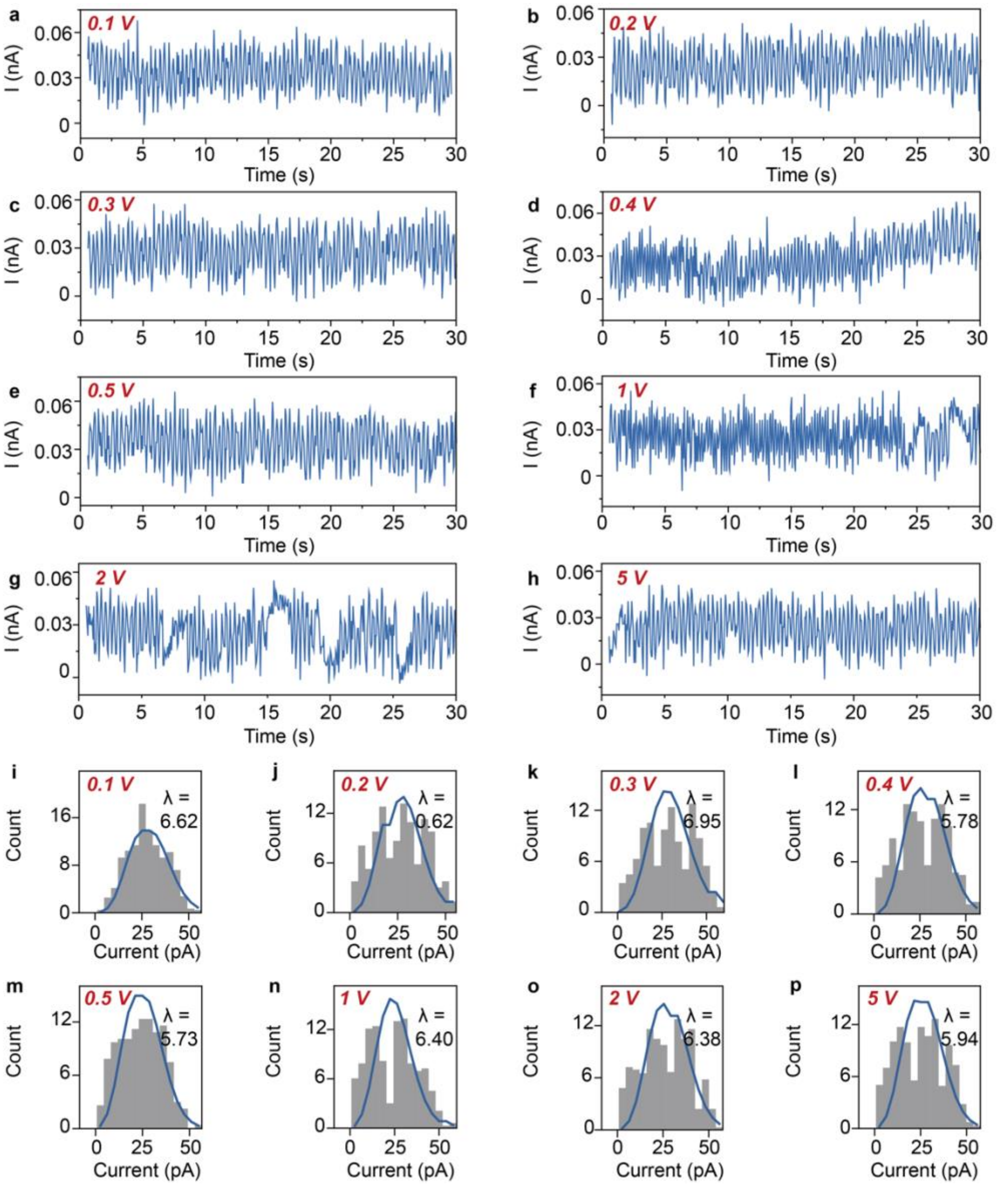

Figure S3. Conductance noise probing at different biases. (a)-(h) Current output of a 1T' $MoTe_2$ device at bias of 0.1 V, 0.2 V, 0.3 V, 0.4 V, 0.5 V, 1 V, 2 V, and 5 V. The temperature is 300 K. (i)-(p) The corresponding histograms and Poisson fittings of the current data points, proving the conductance noise in the 1T' $MoTe_2$ device measured at all the bias conditions is a random process and a reliable physical entropy noise.

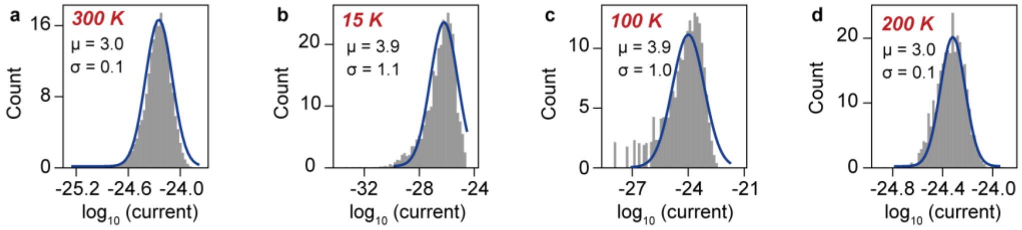

Figure S4. **Semi-log-normal distribution of the 1T' $MoTe_2$ device current output.** Histograms and semi-log-normal distribution fittings of the current outputs from Fig. 2b and c.

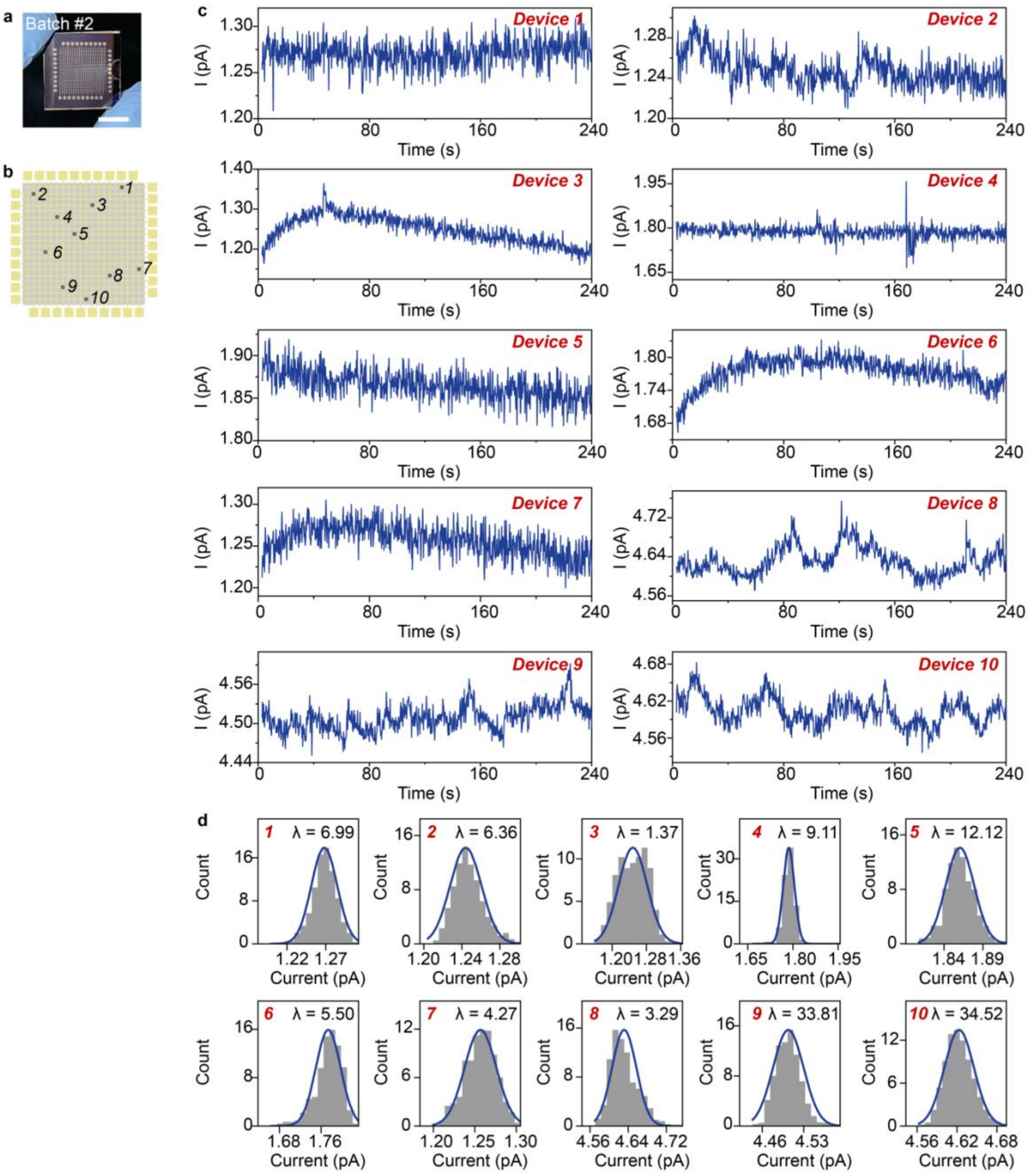

Figure S5. Batch #2 device-to-device conductance noise. (a) 20 ×20 1T' $MoTe_2$ devices, with a fabrication yield of ~70%, and (b) the corresponding schematic device array showing the working devices randomly selected for the sampling test. (c) Current noise as measured from the selected devices, and (d) the corresponding histograms and Poisson fittings of the current data from (c). Scale bar – (a) 1 cm**.**

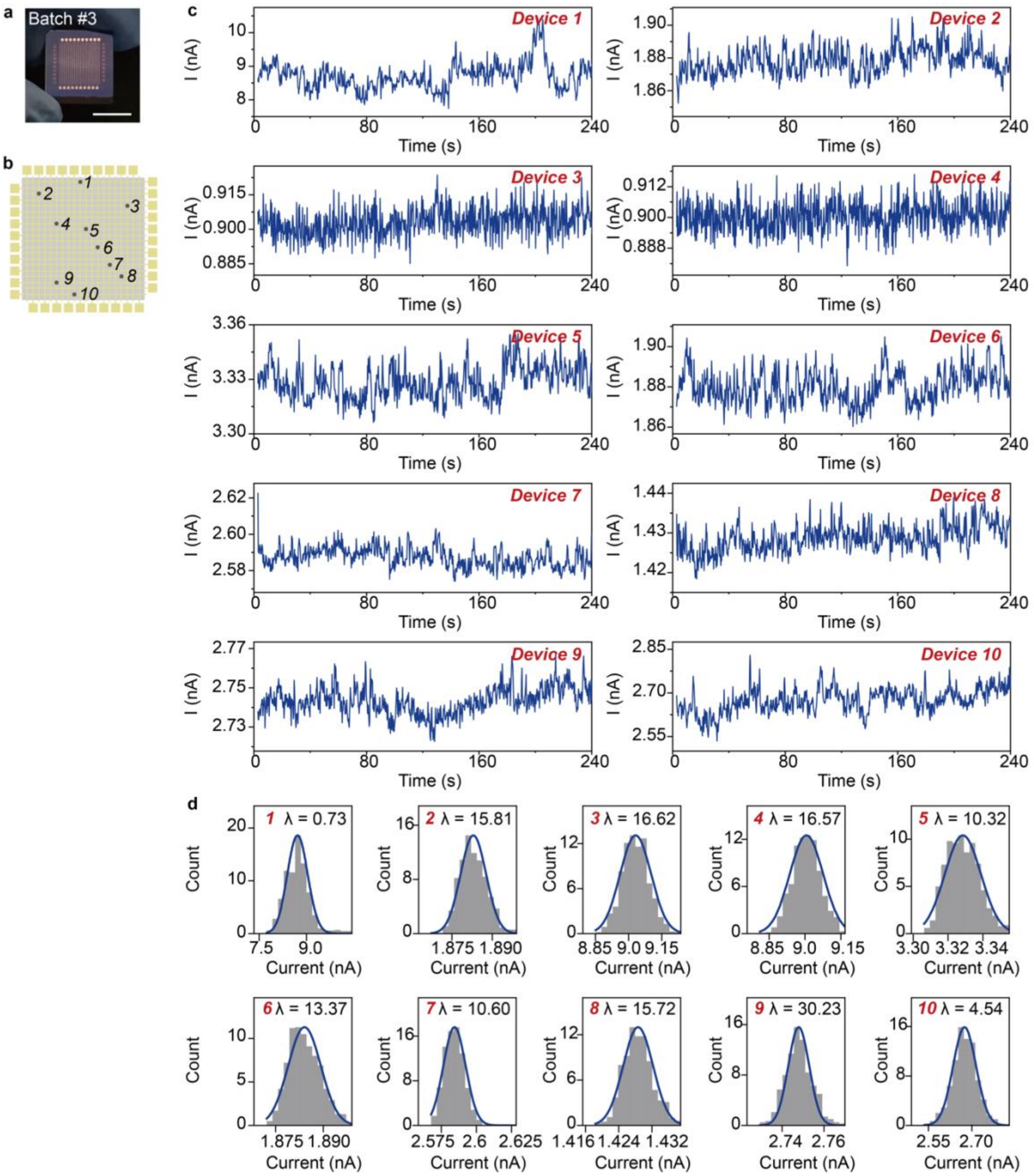

Figure S6. Batch #3 device-to-device conductance noise. (a) 20 ×20 1T' $MoTe_2$ devices, with a fabrication yield of ~80%, and (b) the corresponding schematic device array showing the working devices randomly selected for the sampling test. (c) Current noise as measured from the selected devices, and (d) the corresponding histograms and Poisson fittings of the current data from (c). Scale bar – (a) 1 cm.

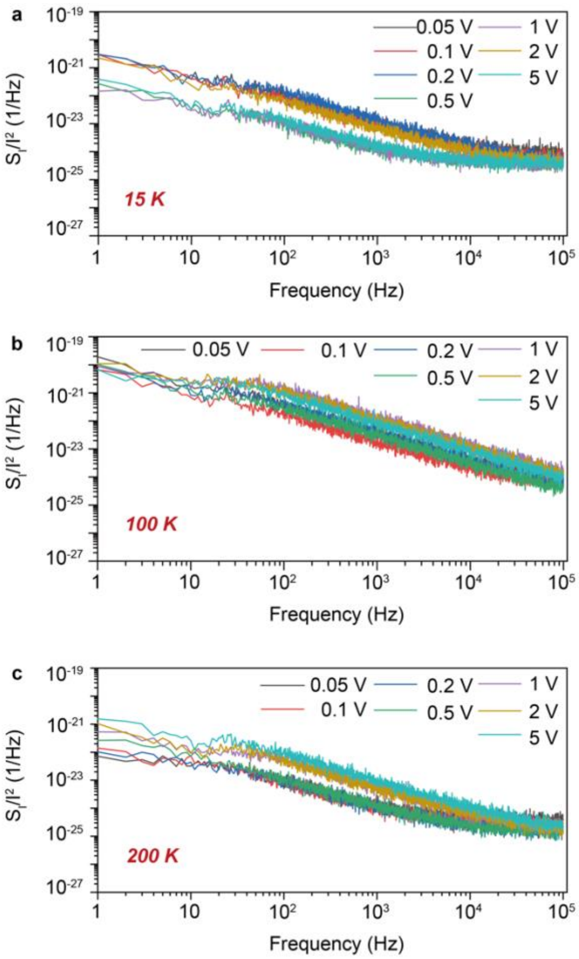

Figure S7. Current power spectral density (PSD) characterization of the 1T' $MoTe_2$ device at low temperatures. The PSD testing is conducted on the 1T' $MoTe_2$ device at a low temperature from 15 K to 200 K at the different bias conditions – (a) 15 K, (b) 100 K, and (c) 200 K. The testing proves 1/f noise in the device for all the temperatures and bias testing conditions in the low-frequency region.

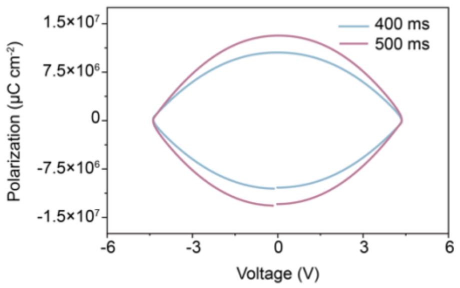

Figure S8. Ferroelectric polarization characterization of the 1T' $MoTe_2$ device. The ferroelectric polarization testing is conducted on a typical 1T' $MoTe_2$ device with a sweeping bias, showing no clear evidence of ferroelectric polarization. The test condition: V_{max} = 5 V; Hysteresis speed = 400 ms/500 ms; Preset delay = 1,000 ms. No significant collective polarization is observed in this testing. Further ferroelectric polarization mapping over individual 1T' $MoTe_2$ nanosheets is required to deterministically locate the exact polarization effect of the ferroelectric dipoles.

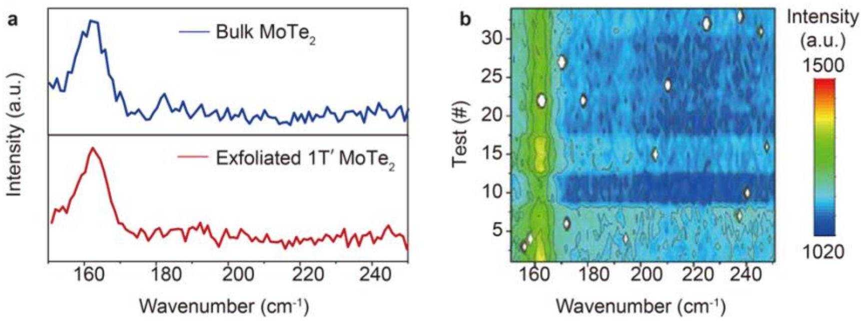

Figure S9. Raman spectroscopy characterization of 1T' $MoTe_2$. (a) Raman spectrum of bulk and the exfoliated 1T' $MoTe_2$. (b) Raman spectrum for the same area taken for 35 times. The test condition: wavelength 532 nm, power 2 mW, integration time for a single test 300s, and spot diameter size 1.5 um. No deterministic Raman peak shifts can be observed. Two main reasons may account for this – 1) as the spot size is 1.5 um in diameter for the Raman spectroscopy characterization, the measured Raman spectrum for the exfoliated 1T' $MoTe_2$ nanosheets is a collective, averaged Raman spectrum of many 1T' $MoTe_2$ nanosheets; and 2) each of the Raman spectroscopy tests takes 300 s, and this long time duration is not able to present the variations of the metastable phase structure and ferroelectric polarization. Further Raman spectroscopy on individual 1T' $MoTe_2$ nanosheets is required to deterministically locate the exact polarization effect of the ferroelectric dipoles.

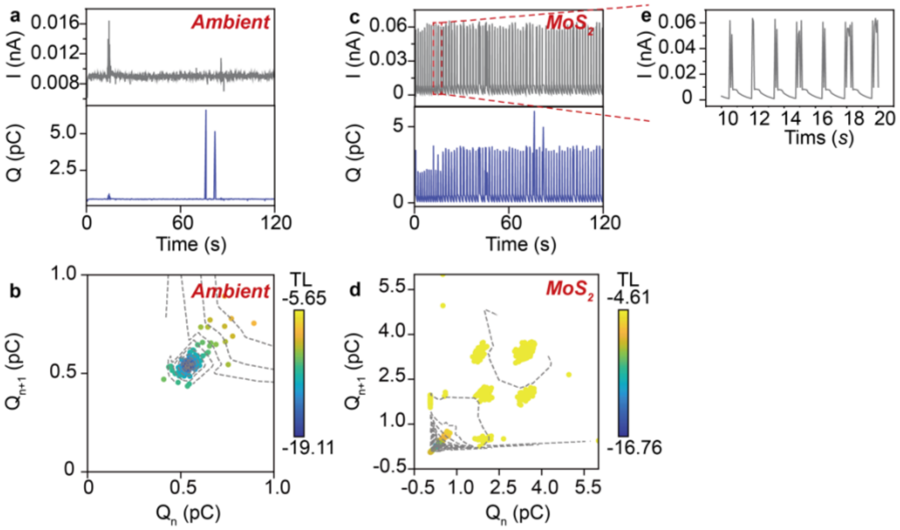

Figure S10. Control experiments of the ambient noise and a MoS_2 device. Current output and the corresponding cumulative charge fluctuations from (a) ambient noise and (c) a MoS_2 device at 0.05 V, 300 K, with (e) showing the detailed current output from the MoS_2 device. Ambient noise is measured by suspending the probing electrodes. MoS_2 is prepared by liquid-phase exfoliation [6], and is then spin-coated and sandwiched between evaporated top and bottom gold electrodes to fabricate the MoS_2 devices. The device fabrication method and the device configuration are the same as that of the 1T' $MoTe_2$ devices. The current output of the MoS_2 device shows a distinct random switching behavior, proving random telegraph noise (RTN) [7] from the MoS_2 device. The origin of the RTN is suggested to be attributed to charge trapping at the defect sites in the MoS_2 material. [8,9] The cumulative charge is integrated during the sampling time interval of 0.067 s. The time-lag plots for the cumulative charge fluctuations of (b) the ambient noise and (d) the MoS_2 device: the $\boldsymbol{TL}$ plot of the ambient noise establishes monostable aggregation with weak correlations in the cumulative charge states, meaning weak correlations in the noise states from the ambient noise; the $\boldsymbol{TL}$ plot of the MoS_2 device establishes random aggregation regions with strong correlations in the corresponding cumulative charge states, which may be a result of the charge trapping and de-trapping mechanism.

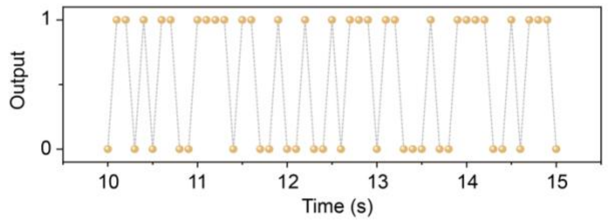

Figure S11. Random bit string of the 0's and 1's true random numbers generated from "*port 4*" during 10 to 15 seconds (the full-time-scale true random number output is shown in Fig. 4c). It is shown that the 0's and 1's true random numbers are generated in a random distribution.

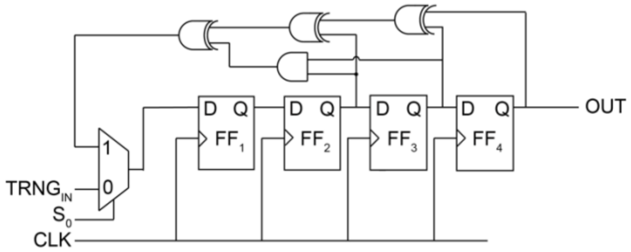

Figure S12. 4-bit nonlinear feedback shift register (NLFSR) circuit design. The NLFSR is used to generate high-throughput random numbers from the seed, i.e. the true random numbers, by setting a high clock frequency, e.g. 1 MHz.

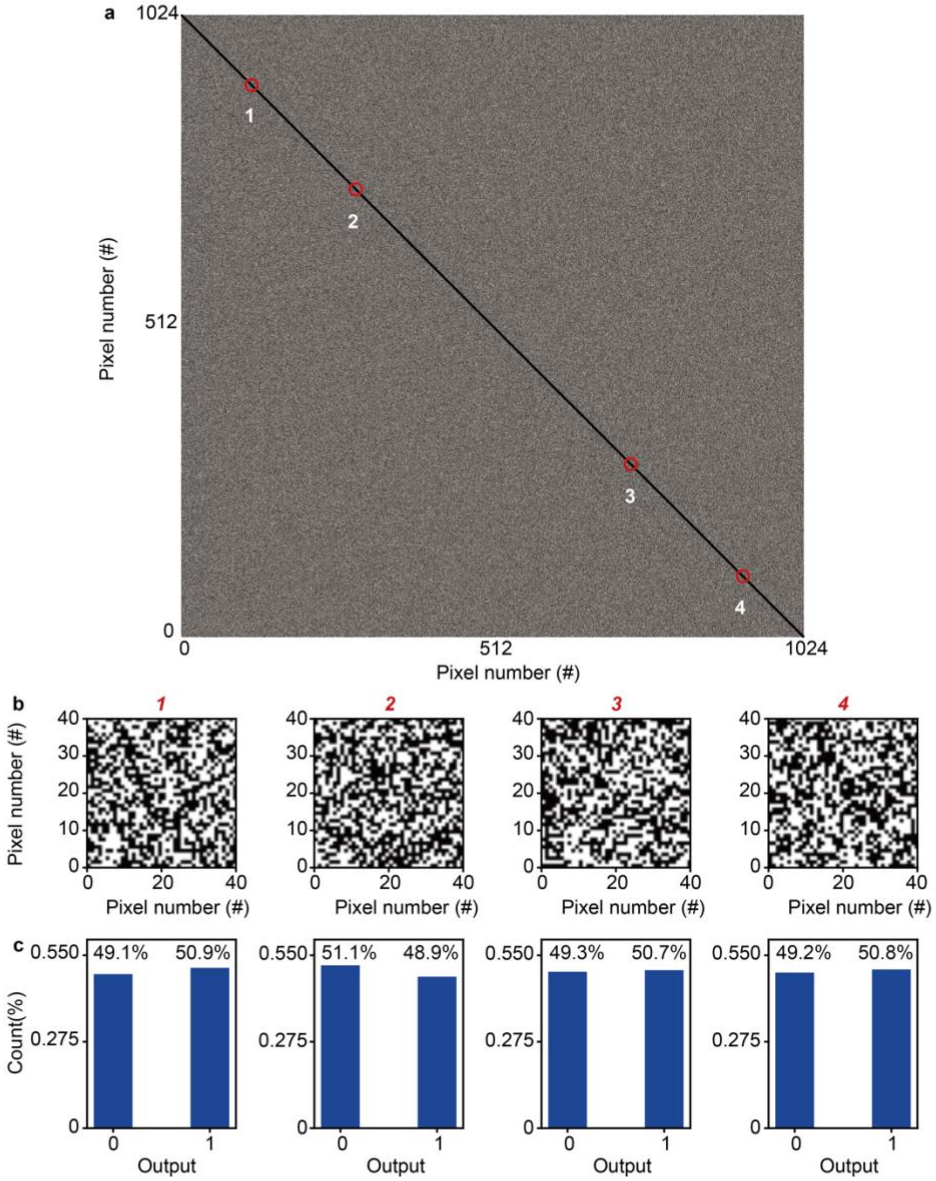

Figure S13. A 1024*1024 bitmap generated using the high-throughput random numbers. To plot the bitmap, the string of the high-throughput random numbers in 0's and 1's digits fills the bitmap sequentially from (0, 0) to (1024, 1024). (a) High-data-volume bitmap. (b) Four randomly selected bitmap regions along the diagonal of the high-data-volume bitmap. (c) The corresponding histograms showing the distributions of the 0's and 1's. The ratios of the 0's and 1's are close to 1 to 1.

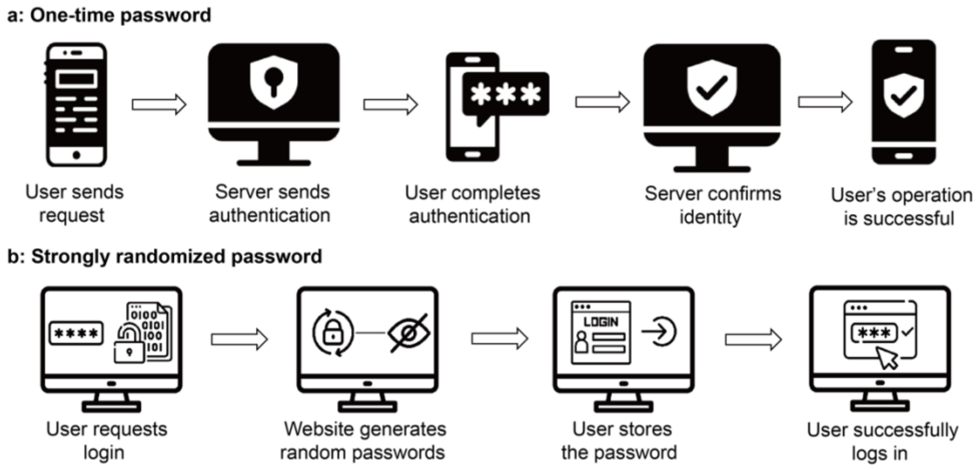

Figure S14. Password generation. (a) Schematic generation and the use of one-time password (OTP) [10]. OTP is a unique password code that is valid for only one login/transaction session. It is often used as a second factor in two-factor authentication (2FA) and multi-factor authentication (MFA) systems [10]. (b) Schematic generation and the use of strongly randomized passwords. Strongly randomized passwords can be stored in systems for applications that require a high level of password security. Please see the generation of the passwords in Supplementary Movie S1.

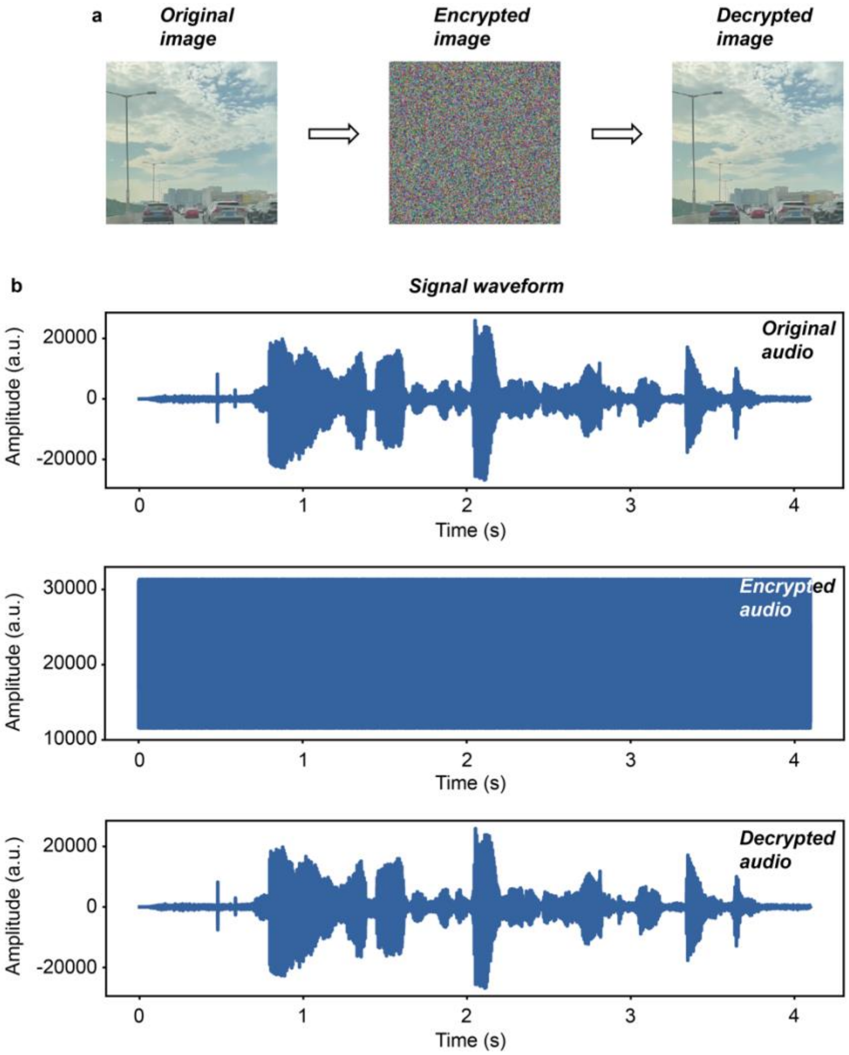

Figure S15. Data encryption. (a) Encryption and decryption of an image as captured in Supplementary Movie S2. The encryption and decryption are conducted by performing the common encryption/decryption operations (e.g. AES encryption/decryption method [11]) on the pixels using the high-throughput random numbers generated. (b) Acoustic spectrograms for audio encryption and decryption, showing a four-second audio, the audio after bit-by-bit encryption, and the audio after decryption. Please see the encryption and decryption of the video and audio in Supplementary Movie S2 and S3. Data credit to YL.

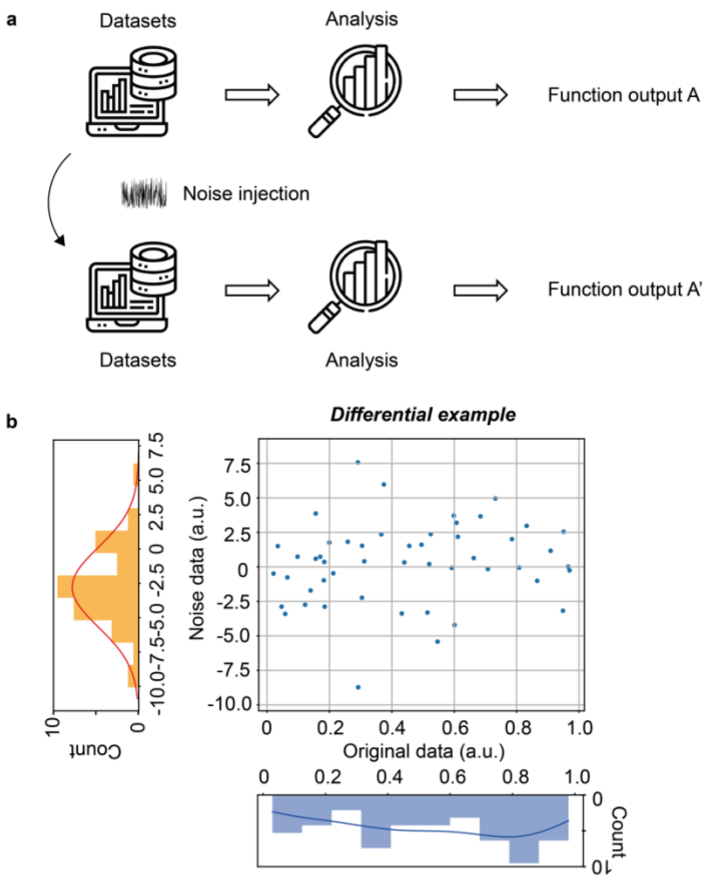

Figure S16. Differential safeguarding strategy. (a) Differential data security workflow. Differential data security is a robust framework used in statistical and machine learning analysis of datasets [12]. The core idea is to ensure that the release of the data (or the statistics derived from the data) does not compromise the security of any individuals in the datasets, and that the datasets with the noise perturbation retain the key features. (b) A case example showing injecting noise to a dataset for data security. The left plot shows the noise data, and the bottom plot the original raw data. The noise data is injected into the original data. An example using the differential protection of biometric information such as facial features in neural networks is presented in Fig. 5.

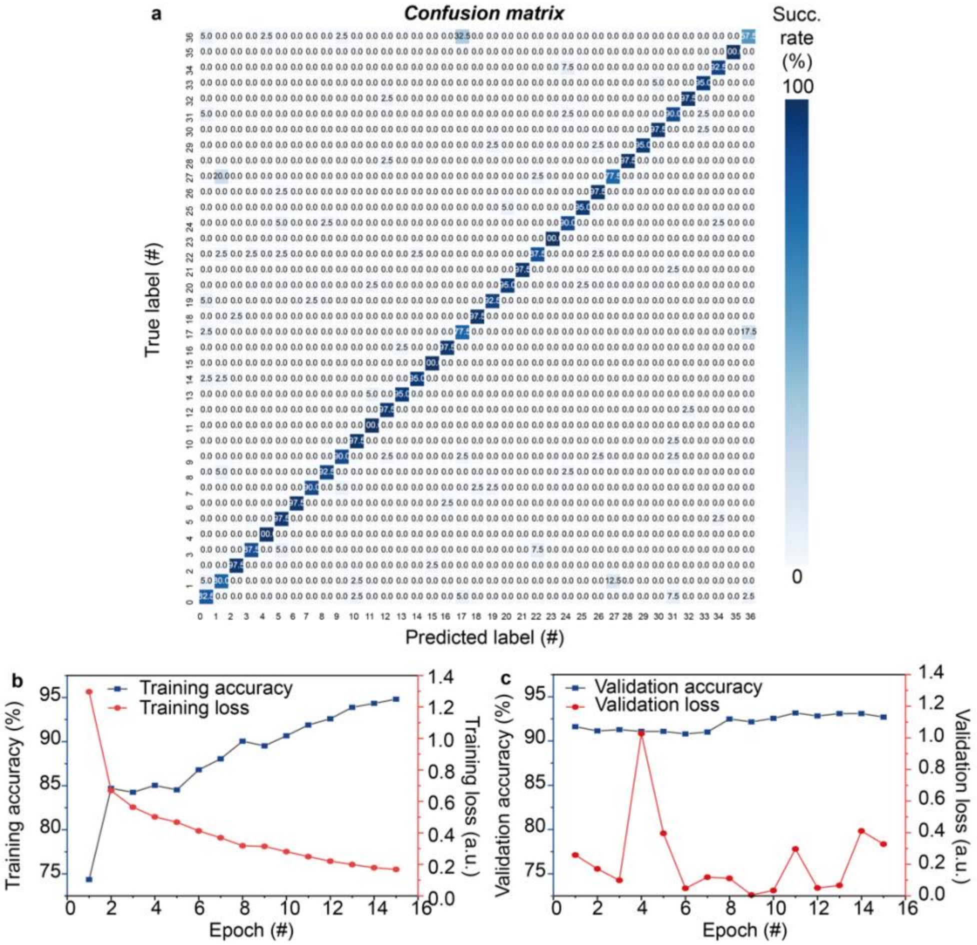

Figure S17. Confusion matrix without noise perturbation and the training performance of the ResNet model. (a) Confusion matrix without noise perturbation. $\boldsymbol{x}$ and $\boldsymbol{y}$ coordinates denote the predicated and true labels of the 37 different classifications in the training dataset, i.e. the Oxford pet-iii dataset. The scale (i.e. the success rate of classification) corresponds to the ratio of the number of the correctly predicted labels to the number of the true labels. The values shown in the confusion matrix represent the success rate, showing the trained ResNet variant achieves a good performance (~92%). (b) The training accuracy and training loss of the ResNet variant. (c) The validation accuracy and validation loss of the ResNet variant. Based on the performance of (b) and (c), it can be concluded that the ResNet variant is well-trained for the Oxford pet-iii dataset.

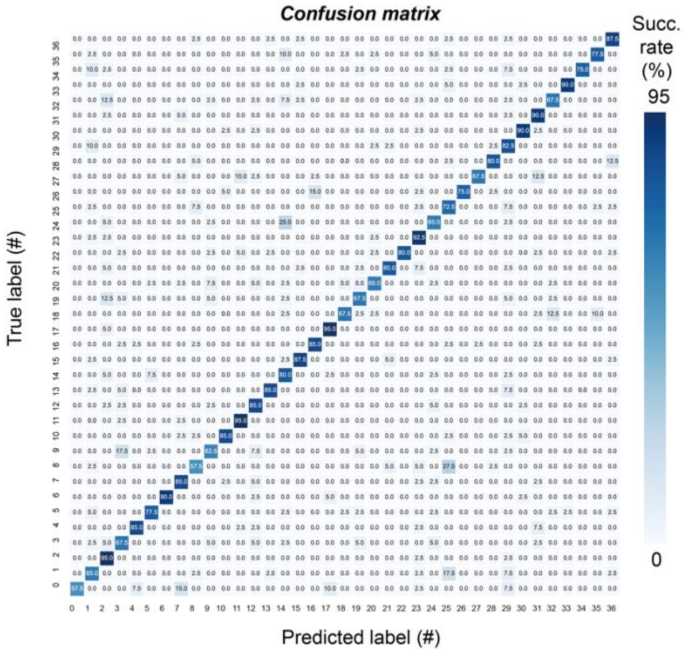

Figure S18. Confusion matrix after noise perturbation, with the success rate values shown. $\boldsymbol{x}$ and $\boldsymbol{y}$ coordinates denote the predicated and true labels of the 37 different classifications in the training dataset, i.e. the Oxford pet-iii dataset. [5] The scale (i.e. the success rate of classification) corresponds to the ratio of the number of the correctly predicted labels to the number of the true labels. The values shown in the confusion matrix represent the success rate. Comparison with the confusion matrix in Fig. S17a proves that the noise perturbation reduces the accuracy of the ResNet variant model.

Supplementary Tables

Table S1. NIST test of the true random numbers.

	P-value	***Success***	***Post-processing***
Approximate entropy	1.0	Success	No
Block frequency	0.824	Success	No
Cumulative sums	0.728, 0.526	Success	No
FFT	0.041	Success	No
Frequency	0.823	Success	No
Linear complexity	-	-	Limited throughput
Longest run	0.445	Success	No
Non-overlapping template	0.999	Success	No
Overlapping template	-	-	Limited throughput
Random excursions	-	Success	No
Random excursions variant	-	Success	No
Rank	-	-	Limited throughput
Runs	0.017	Success	No
Serial	0.499, 0.499	Success	No
Universal	-	-	Limited throughput

Table S2. NIST test of the high-throughput random numbers.

	P-value	*Proportion*	*Success*	*Post-processing*
Approximate entropy	0.409	1	Success	No
Block frequency	0.373	1	Success	No
Cumulative sums	0.047	0.97	Success	No
FFT	0.500	1	Success	No
Frequency	0.057	0.98	Success	No
Linear complexity	0.371	1	Success	No
Longest run	0.965	1	Success	No
Non-overlapping template	0.999	1	Success	No
Overlapping template	0.873	1	Success	No
Random excursions	0.525	1	Success	No
Random excursions variant	0.428	1	Success	No
Rank	0.696	0.99	Success	No
Runs	0.630	1	Success	No
Serial	0.607	1	Success	No
Universal	0.425	1	Success	No

Table S3. Comparison between our random number generator and other reported random number generators.

Material and device	Working principle	Circuit complexity	Integration	Exacting difficulty	Power consumption	Temperature window (K)	Applications	Ref.
Metal/Oxide/Metal memristors	RTN in RRAM	Moderate	high	Moderate	0.18-18,900 nW	295.15	TRNG	13
Pt/Ag/Ag:SiO_2/Pt	Stochastic switching in RRAM	Low	high	Easy	/	295.15-358.15	TRNG	14
Pt/Ti/NbO_x/Pt	Stochastic oscillation in RRAM	Moderate	high	Easy	/	300-390	TRNG	15
Silicon-based MTJ	Stochastic switching time in MRAM	High	Moderate	Difficult	18 pJ/bit	248-373.15	TRNG	16
MTJ	Stochastic magnetization in MRAM	/	Moderate	Difficult	/	300	TRNG	17
MTJ with CNTFET	Stochastic behavior in MRAM	High	Moderate	Difficult	1.11 pJ/bit	300	TRNG	18
Silicon-based FeFET	Process variations and ferroelectric-material degradation in FeRAM	High	Moderate	Moderate	/	300	TRNG	19
TiN/HfO_2/SiON FeFET	Stochastic switching in FeRAM	Low	Moderate	Moderate	/	/	TRNG	20

Straintronic GFET	Strain-induced reversible cracking	/	/	Easy	/	298.15	PUF	21
MoS_2 FET	Inherent stochasticity in the voltage transfer	Low	Moderate	Easy	30 pJ/bit	298.15-373.15	TRNG	22
MoS_2 memtransistor	D2D variation	/	/	Easy	/	298.15	PUF	23
TMO/TMD camouflaged resistors	the properties of TMDs and transition-metal oxides	/	/	Easy	/	/	Hardware obfuscation	24
MoS_2 memtransistor	Programming stochasticity	Low	/	Easy	<1 nJ	/	Stochastic computing	25
GFET	D2D variation	/	/	Easy	/	100-380	PUF	26
hBN memristor	RTN in RRAM	Moderate	high	Easy	/	295.15-423.15	TRNG	27
**This work**	**Polarization variation**	**Low**	**High**	**Easy**	**50 nW**	**15-300**	**TRNG**	/

Note the power consumption in this table considers the consumption from the functional device only. (MTJ: magnetic tunnel junction; CNTFET: carbon nanotube field-effect transistor; FeFET: ferroelectric field-effect transistor; GFET: graphene field-effect transistor; FET: field-effect transistor; TMO/TMD: transition metal oxide/transition metal dichalcogenide; RTN: random telegraph noise; RRAM: resistive random-access memory; MRAM: magnetoresistive random-access memory; FeRAM: ferroelectric random-access memory; D2D: device-to-device; TRNG: true random number generator; PUF: physical unclonable function.)

Supplementary References

(1) Yu, W.; Dong, Z.; Abdelwahab, I.; Zhao, X.; Shi, J.; Shao, Y.; Li, J.; Hu, X.; Li, R.; Ma, T.; Wang, Z.; Xu, Q.-H.; Tang, D. Y.; Song, Y.; Loh, K. P. High-Yield Exfoliation of Monolayer 1T'-$MoTe_2$ as Saturable Absorber for Ultrafast Photonics. *ACS Nano* **2021**, *15* (11), 18448–18457.

(2) Modine, F. A.; Major, R. W.; Choi, S. I.; Bergman, L. B.; Silver, M. N. Polarization Currents in Varistors. *J. Appl. Phys.* **1990**, *68* (1), 339–346.

(3) Hartke, J. L. The Three-dimensional Poole-Frenkel Effect. *J. Appl. Phys.* **1968**, *39* (10), 4871–4873.

(4) He, K.; Zhang, X.; Ren, S.; Sun, J. Deep Residual Learning for Image Recognition. In *2016 IEEE Conference on Computer Vision and Pattern Recognition (CVPR)*, Las Vegas, NV, USA, 2016, pp. 770–778.

(5) Parkhi, O. M.; Vedaldi, A.; Zisserman, A.; Jawahar, C. Cats and Dogs. In *2012 IEEE conference on computer vision and pattern recognition (CVPR)*, Providence, RI, USA, 2012, pp. 3498–3505.

(6) Hu, G.; Yang, L.; Yang, Z.; Wang, Y.; Jin, X.; Dai, J.; Wu, Q.; Liu, S.; Zhu, X.; Wang, X.; Wu, T.-C.; Howe, R. C. T.; Albrow-Owen, T.; Ng, L. W. T.; Yang, Q.; Occhipinti, L. G.; Woodward, R. I.; Kelleher, E. J. R.; Sun, Z.; Huang, X.; Zhang, M.; Bain, C. D.; Hasan, T. A General Ink Formulation of 2D Crystals for Wafer-Scale Inkjet Printing. *Sci. Adv.* **2020**, *6* (33), eaba5029.

(7) Kogan, S. Random Telegraph Noise in Microstructures. *Phys. Rev. Lett.* **1998**, *81* (14), 2986.

(8) Song, S. H.; Joo, M.-K.; Neumann, M.; Kim, H.; Lee, Y. H. Probing Defect Dynamics in Monolayer MoS_2 via Noise Nanospectroscopy. *Nat. Commun.* **2017**, *8* (1), 2121.

(9) Ma, X.; Liu, Y.-Y.; Zeng, L.; Chen, J.; Wang, R.; Wang, L.-W.; Wu, Y.; Jiang, X. Defects Induced Charge Trapping/Detrapping and Hysteresis Phenomenon in MoS_2 Field-Effect Transistors: Mechanism Revealed by Anharmonic Marcus Charge Transfer Theory. *ACS Appl. Mater. Interfaces* **2022**, *14* (1), 2185–2193.

(10) Ometov, A.; Bezzateev, S.; Mäkitalo, N.; Andreev, S.; Mikkonen, T.; Koucheryavy, Y. Multi-Factor Authentication: A Survey. *Cryptography* **2018**, *2* (1), 1.

(11) National Institute of Standards and Technology (NIST); Dworkin, M. J.; Barker, E.; Nechvatal, J.; Foti, J.; Bassham, L. E.; Roback, E.; Dray, J. Advanced Encryption Standard (AES); Federal Inf. Process. Stds. (NIST FIPS), 2001, 197.

(12) Dwork, C. Differential Privacy. In *Automata, Languages and Programming*; Bugliesi, M., Preneel, B., Sassone, V., Wegener, I., Eds.; Springer Berlin Heidelberg: Berlin, Heidelberg, 2006; pp. 1–12.

(13) Li, X.; Zanotti, T.; Wang, T.; Zhu, K.; Puglisi, F. M.; Lanza, M. Random Telegraph Noise in Metal-Oxide Memristors for True Random Number Generators: A Materials Study. *Adv. Funct. Mater.* **2021**, *31* (27), 2102172.

(14) Jiang, H.; Belkin, D.; Savel'ev, S. E.; Lin, S.; Wang, Z.; Li, Y.; Joshi, S.; Midya, R.; Li, C.; Rao, M.; Barnell, M.; Wu, Q.; Yang, J. J.; Xia, Q. A Novel True Random Number Generator Based on a Stochastic Diffusive Memristor. *Nat. Commun.* **2017**, *8* (1), 882.

(15) Kim, G.; In, J. H.; Kim, Y. S.; Rhee, H.; Park, W.; Song, H.; Park, J.; Kim, K. M. Self-Clocking Fast and Variation Tolerant True Random Number Generator Based on a Stochastic Mott Memristor. *Nat. Commun.* **2021**, *12* (1), 2906.

(16) Yang, K.; Dong, Q.; Wang, Z.; Shih, Y.-C.; Chih, Y.-D.; Chang, J.; Blaauw, D.; Svlvester, D. A 28NM Integrated True Random Number Generator Harvesting Entropy from MRAM. *2018 IEEE Symposium on VLSI Circuits (VLSIC)*, Honolulu, HI, USA, 2018, pp. 171–172.

(17) Chen, X.; Zhang, J.; Xiao, J. Magnetic-Tunnel-Junction-Based True Random-Number Generator with Enhanced Generation Rate. *Phys. Rev. Appl.* **2022**, *18* (2), L021002.

(18) Amirany, A.; Jafari, K.; Moaiyeri, M. H. True Random Number Generator for Reliable Hardware Security Modules Based on a Neuromorphic Variation-Tolerant Spintronic Structure. *IEEE Trans. Nanotechnol.* **2020**, *19*, 784–791.

(19) Siu, J. W. K.; Eslami, Y.; Sheikholeslami, A.; Gulak, P. G.; Endo, T.; Kawashima, S. A Current-Based Reference-Generation Scheme for 1T-1C Ferroelectric Random-Access Memories. *IEEE J. Solid-State Circuits* **2003**, *38* (3), 541–549.

(20) Mulaosmanovic, H.; Mikolajick, T.; Slesazeck, S. Random Number Generation Based on Ferroelectric Switching. *IEEE Electron Device Lett.* **2017**, *39* (1), 135–138.

(21) Ghosh, S.; Zheng, Y.; Radhakrishnan, S. S.; Schranghamer, T. F.; Das, S. A Graphene-Based Straintronic Physically Unclonable Function. *Nano Lett.* **2023**, *23* (11), 5171–5179.

(22) Ravichandran, H.; Sen, D.; Wali, A.; Schranghamer, T. F.; Trainor, N.; Redwing, J. M.; Ray, B.; Das, S. A Peripheral-Free True Random Number Generator Based on Integrated Circuits Enabled by Atomically Thin Two-Dimensional Materials. *ACS Nano* **2023**, *17* (17), 16817–16826.

(23) Oberoi, A.; Dodda, A.; Liu, H.; Terrones, M.; Das, S. Secure Electronics Enabled by Atomically Thin and Photosensitive Two-Dimensional Memtransistors. *ACS Nano* **2021**, *15* (12), 19815–19827.

(24) Wali, A.; Ravichandran, H.; Das, S. A Machine Learning Attack Resilient True Random Number Generator Based on Stochastic Programming of Atomically Thin Transistors. *ACS Nano* **2021**, *15* (11), 17804–17812.

(25) Ravichandran, H.; Zheng, Y.; Schranghamer, T. F.; Trainor, N.; Redwing, J. M.; Das, S. A Monolithic Stochastic Computing Architecture for Energy Efficient Arithmetic. *Adv. Mater.* **2023**, *35* (2), 2206168.

(26) Dodda, A.; Subbulakshmi Radhakrishnan, S.; Schranghamer, T. F.; Buzzell, D.; Sengupta, P.; Das, S. Graphene-Based Physically Unclonable Functions That Are Reconfigurable and Resilient to Machine Learning Attacks. *Nat. Electron.* **2021**, *4* (5), 364–374.

(27) Wen, C.; Li, X.; Zanotti, T.; Puglisi, F. M.; Shi, Y.; Saiz, F.; Antidormi, A.; Roche, S.; Zheng, W.; Liang, X.; Hu, J.; Duhm, S.; Roldan, J. B.; Wu, T.; Chen, V.; Pop, E.; Garrido, B.; Zhu, K.; Hui, F.; Lanza, M. Advanced Data Encryption Using 2D Materials. *Adv. Mater.* **2021**, *33* (27), 2100185.
